# The effect of COVID-19 home quarantine on the psychological state of pharmacy students: a cross-sectional study

**DOI:** 10.1186/s40545-022-00472-6

**Published:** 2022-11-04

**Authors:** Asmaa S. Mohamed, Ahmed A. Abdelrahman, Hosam M. Ahmad, Omar R. Abdel Aziz, Yasmeen S. Mesameh, Soad A. Mohamad

**Affiliations:** 1 Clinical Pharmacy Department, Faculty of Pharmacy, Port Said University, Port Said, 42526 Egypt; 2 Neuropsychiatry Department, Faculty of Medicine, Assiut University, Assiut, 71515 Egypt; 3 Internal Medicine and Biomedical Chemistry Departments, Egypt Ministry of Health and Population, Minya, 61516 Egypt; 4 Faculty of Pharmacy, Deraya University, Minya, Egypt; 5 Pharmaceutics and Clinical Pharmacy Department, Faculty of Pharmacy, Deraya University, Minya, Egypt

**Keywords:** COVID-19, Depression, Quarantine, Students, Mental health, Pharmacy

## Abstract

**Background:**

Psychological morbidity has been documented in medical and pharmaceutical undergraduate students in different countries around the world. In this study, we examined the impact of coronavirus disease 2019 (COVID-19) home quarantine on the depressive psychological aspects of last-grade pharmacy students.

**Methods:**

A cross-sectional study was conducted by the Department of Clinical Pharmacy, Faculty of Pharmacy, Deraya University, Egypt. Two hundred and sixty-eight last-grade pharmacy students were included in this study, and they completed a self-administered, pre-designed, anonymous questionnaire. The main outcome measures were the Hamilton Depression Rating Scale (HRS) and Patient Health Questionnaire-9 (PHQ-9), which were measured to screen for the symptoms of psychological depression and determine the degree of depression severity between the beginning and the end of the COVID-19 home quarantine period. Data entry and analysis were done using the Statistical Package for Social Science (SPSS) software version 26. Descriptive statistics were employed for analyses of the data, and categorical variables were described by frequencies and percentages. Bivariate and multivariable analyses were performed to examine relations between demographic data and psychological scales. The study protocol was approved by the Faculty of Pharmacy, Minia University Ethical Committee.

**Results:**

A total of 268 students participated in this study (102 males and 166 females). The mean ± SD score of baseline HRS and HRS at the end of the study was 6.3 ± 4.45, 7.95 ± 5.36, respectively, with the presence of a statistically significant difference between the two scores (*p* < 0.001). The mean ± SD score of baseline PHQ-9 and PHQ-9 at the end of the study was 4.35 ± 3.45, 5.37 ± 4.14, respectively, with the presence of a statistically significant difference between the two scores (*p* < 0.001). The results showed that the COVID-19 home quarantine period led to a depressive psychological effect on the students in this study.

**Conclusions:**

Students’ psychological depression causes morbidity and, in some cases, mortality. Psychological depressive problems were significantly associated with the COVID-19 home quarantine period, which calls for early intervention to solve it. Student counselling services must be more accessible and affordable to overcome this problem.

## Background

The outbreak of coronavirus disease (COVID-19) created a public health emergency of international concern [1]. WHO assumed different options to prevent the introduction of the disease to new areas or to reduce human-to-human transmission, like quarantine, which means limiting the movement of people to decrease exposure to the virus [2]. Quarantine was implemented successfully as an effective measure during the SARS epidemic in 2003 [3].

One of the COVID-19 quarantine drawbacks is the restriction of online learning for all grades, including university. Pharmacy students are one of these groups who were asked to complete their curriculum with the aid of online sources. Psychological disorders in these students negatively affect their future, leading to decreased quality of life, lower output, and learning difficulties [4, 5]. The environment of medical colleges was considered extremely stressful and so they have been an issue of concern for many researchers [6–9]. Medical students are a characteristic group of people that are very sensitive and trying to fit in, maintain good grades, plan for the future. Being in quarantine away from their colleague leads to higher academic disorder, inability to manage, and may cause anxiety for many students [10]. Stress may make some students get depressed. Starting a new experiment as online learning, especially in the year of graduation can increase this depression that may cause severe problems [11]. Several studies measure depression resulting from quarantine [1, 12, 13] and all of them approved that depression is one of the signs of quarantine. Other signs such as depression, anorexia, guilt, and agitation are among serious conditions of low mood and distaste for activity that can affect an individual’s thoughts, behavior, feelings, and sense of well-being. If long-lasting and with moderate or severe intensity, these problems may become a serious health condition. It can cause severe suffering and a poor job at work, school, and home. At its worst, that can lead to suicide [14, 15].

While the studies mentioned above provide valuable information about how COVID-19 home quarantine/pandemic isolation affects the psychological state of the students, the results may be different according to the country, society, type of the college, and grade of the students, the study focuses on the pharmacy students’ last year. The novelty of this study lies in using two different psychological scales of depression The importance of this study lies in emphasizing the psychological effects of the COVID-19 home quarantine period on the students and its relation to some demographic data that may be modifiable to some extent, which helps to prevent negative psychological effects on the students.

The purpose of this study is to estimate the presence of depression among pharmacy students at Deraya University following the end of the COVID-19 home quarantine period, as well as the impact of this isolation on their psychological health state, by comparing previous data collected at the start of home quarantine using the Hamilton Depression Rating Scale (HRS) and Patient Health Questionnaire-9 (PHQ-9) [16, 17].

## Methods

### Study design, population, and setting

**Fig. 1:**
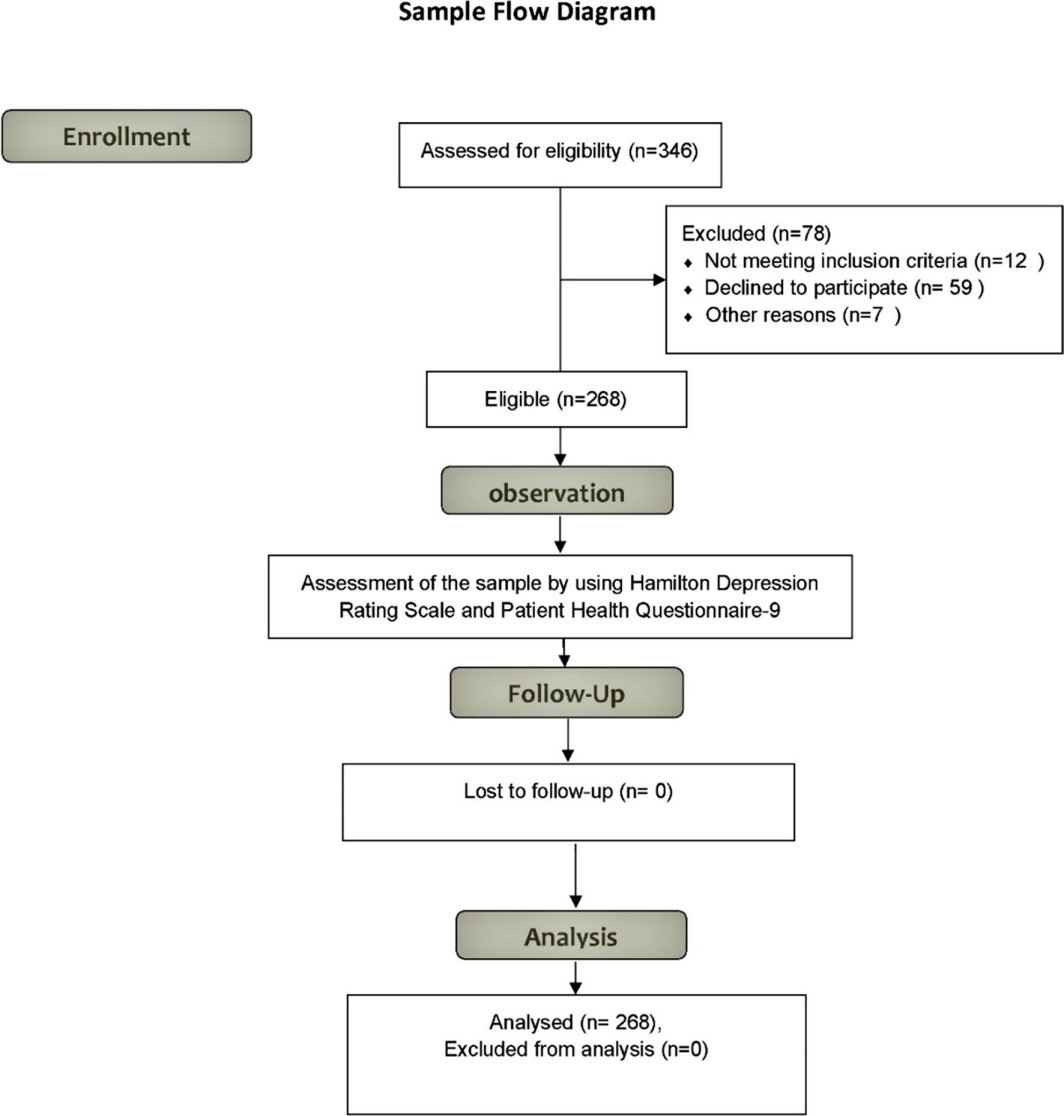
Flow diagram of the studied sample

A retrospective cross-sectional study was conducted in the Pharmacy College at Deraya University in Egypt for 3 months. Last-grade pharmacy students at Deraya University were selected as participants. Calculation of sample size. The minimal sample size was calculated using a sample size calculator program with a 95% confidence level and 0.05 standard error. Out of the pharmacy students’ last grade (*n* = 346), the enrollment was 346 students, but 268 students participated and completed the questionnaires (as shown in Fig. 1).

Students who failed to fulfill all inclusion and exclusion criteria were excluded from the study. The inclusion criteria for this study were as follows: full-time undergraduate students in the last grade who were ≥ 22 years of age; providing valid oral and written consent; and comprehending the questionnaire. Participants with pregnancy, a malignant tumor, a mental or psychiatric disorder, or any disease that can affect the psychological or nervous state were excluded from the present study.

Data were collected on socio-demographic characteristics. Sleep and screen time were determined by asking “on average, how many hours of sleep do you usually have during the daytime and night?” and “how many hours per day do you watch TV, computer, or mobile?” physical activity index [18]. The total score for the physical activity index is the sum of multiplying intensity × duration × frequency of an exercise. The score of (81 to 100) = very active lifestyle, (60 to 80) = active and healthy; (40 to 59) = acceptable but could be better; (20 to 39) = not good enough; (Under 20) = sedentary. A self-administered, pre-designed, anonymous questionnaire based on the Hamilton Depression Rating Scale (HRS) and Patient Health Questionnaire-9 (PHQ-9) had been filled out by the students.

The outcome variables are: a simple questionnaire was selected to cover all the data required in this study. It was used to screen for the severity of depression. This questionnaire was self-reported by the same participants at the beginning and at the end of the COVID-19 home quarantine period for 3 months by using electronic election through Microsoft Team software. The scales that were used in this study have been validated by much research [19, 20]. The HRS is designed to rate the severity of depression in patients. The severity of depression was classified as follows: normal = (0–7), mild depression = (8–13), moderate depression = (14–18), severe depression = (19–22), and very severe depression = (> 23). The PHQ-9 is a self-administered questionnaire that measures the degree of depression severity. The severity of depression was classified as follows: depression can be classified as mild (0–9), moderate (10–14), moderately severe (15–19), or severe (20–27).

### Data analysis

The Statistical Package for the Social Sciences (SPSS) version 26 statistical software was used to enter and analyze data. Results are presented as counts and percentages for categorical variables, means and standard deviation (SD), or median and range, as appropriate for continuous variables. A paired *t*-test was used to compare the students’ scores on the scale. The independent *t*-test was used to determine whether there is a statistically significant difference between the means of two unrelated groups. The one-way analysis of variance (ANOVA) is used to determine whether there are any statistically significant differences between the means of two or more independent (unrelated) groups. *p*-value < 0.05 was set as the threshold of statistical significance.

## Results

Out of 346 final-grade pharmacy students, 268 participated and completed the questionnaires. This study showed that the overall mean age was 22.99 ± 0.84 years, 166 (61.9%) were females, (89.9%) with married parents, (88.1%) were urban, (55.2%) with no special habits, and (81%) with a family monthly income of more than 5000 Egyptian pounds. (92.5%) with sedentary physical index (76.9%) and more than 6 h of screen time per day, which includes TV, computers, video games, and mobile phones. Regarding sleeping time (49.6%) with ≤ 6 h of sleeping per day, (56.72%) with ≤ 4 h per day studying time (as shown in Table 1).

**Table 1:** Demographic data of the studied sample

Baseline characteristics	Number	%	Baseline characteristics	Number	%
*Age*			*Physical index*		
Range	22–24 y		Sedentary	248	92.5
Mean ± standard deviation	22.99 ± 0.84		Not good enough	20	7.5
*Gender*			*Screen time*		
Male	102	38.1	≤ 4H/D	22	8.2
Female	166	61.9	4–6 H/D	40	14.9
			> 6 H/D	206	76.9
*Parental marital status*			*Sleep time*		
Married	241	89.9	> 8 H/D	38	14.2
Divorced	15	5.6	6–8 H/D	97	36.2
Live separate or widowed	12	4.5	≤ 6 H/D	133	49.6
*Residence*			*Family monthly income*		
Urban	236	88.1	< 5000 E.P	51	19
Rural	32	11.9	> 5000 E.P	217	81
*Special habits*			*Studying time*		
Tea or coffee > 3 times/day	106	39.6	≤ 4 H/D	152	56.72
None	148	55.2	4–6 H/D	73	27.24
Smoking	14	5.2	> 6 H/D	43	16.04

*H/D* hour per day, *E.P.* Egyptian pound

The difference between the depression scales (Hamilton Rating Scale and Patient Health Questionnaire-9) of the students at the beginning of the study was significant according to gender (female score > male), family income (the higher the income, the lower the score), parental marital status (stable marital status had the lowest score), physical index (sedentary index had more score), and sleep time (best score when sleep time was between 6–8 H/D). There was no significant difference according to residence, special habits, screen time, studying time, and last academic performance (as shown in Table 2).

**Table 2:** Relation between demographic data and both Hamilton Rating Scale and Patient Health Questionnaire-9 at the beginning of the study

	*n*	Hamilton Rating Scale		Patient Health Questionnaire-9	
		Mean ± st. deviation	*p* value	Mean ± st. deviation	*p* value
*Gender*					
Male	102	5.33 ± 4.317	**0.005**	3.74 ± 3.279	**0.02**
Female	166	6.90 ± 4.429		4.73 ± 3.510	
*Family income*					
< 5000 E.P	51	11.55 ± 3	**< 0.001**	7.80 ± 2.706	**< 0.001**
> 5000 E.P	217	5.07 ± 3.78		3.54 ± 3.093	
*Parental marital status*					
Married	241	5.79 ± 4.07	**< 0.001**	3.99 ± 3.251	**< 0.001**
Divorced, separated or widowed	27	10.85 ± 5.14		7.59 ± 3.565	
*Residence*					
Urban	236	6.34 ± 4.48	0.71	4.44 ± 3.512	0.25
Rural	32	6.03 ± 4.27		3.69 ± 2.934	
*Physical index*					
Sedentary	248	6.56 ± 4.491	**< 0.001**	4.55 ± 3.497	**< 0.001**
Not good enough	20	3.29 ± 2.305		2 ± 1.5	
*Special habits*					
Tea or coffee > 3 times/D	106	6.45 ± 4.76	0.45	4.46 ± 3.87	0.62
Smoking	14	4.86 ± 2.958		3.50 ± 2.21	
None	148	6.33 ± 4.323		4.36 ± 3.23	
*Screen time*					
< 4 H/D	22	6.5 ± 4.47	0.13	4.5 ± 2.91	0.69
4–6 H/D	40	5 ± 3.35		3.93 ± 2.65	
> 6 H/D	206	6.53 ± 4.6		4.42 ± 3.64	
*Sleep time*					
< 6 H/D	38	11.47 ± 3.58	**< 0.001**	7.53 ± 3.25	**< 0.001**
6–8 H/D	97	3.78 ± 2.84		2.80 ± 1.99	
> 8 H/D	133	6.66 ± 4.24		4.58 ± 3.68	
*Studying time*					
< 4 H/D	152	6.66 ± 4.52	0.16	4.55 ± 3.6	0.34
4–6 H/D	73	6.22 ± 4.49		4.34 ± 3.43	
> 6 H/D	43	5.19 ± 3.99		3.67 ± 2.89	
*Last academic performance*					
Acceptable	36	7.28 ± 5.012	0.07	5.58 ± 4.33	0.08
Good	34	5.24 ± 3.285		3.74 ± 2.34	
Very good	86	6.97 ± 4.731		4.48 ± 3.64	
Excellent	112	5.80 ± 4.242		4.05 ± 3.2	

Bold values are significant

*H/D* hour per day

There is a significant difference between depression scores at the beginning [HRS score (0), PHQ9 score (0)] and at the end [HRS score (1), PHQ9 score (1)] of the study (COVID-19 quarantine period), where at the end of the study the scores were higher than at the beginning of the study (as shown in Table 3).

**Table 3:** Hamilton Rating Scale and Patient Health Questionnaire-9 before and after COVID-19 home quarantine period

Hamilton Rating Scale			
	HRS Score (0)	HRS Score (1)	*p*-value
Mean ± std. deviation	6.3 ± 4.45	7.95 ± 5.36	**< 0.001**

Patient Health Questionnaire-9			
	PHQ9 score (0)	PHQ9 score (1)	*p*-value
Mean ± std. deviation	4.35 ± 3.45	5.37 ± 4.14	**< 0.001**

Bold values are significant

HRS Score (0) = Hamilton Rating Scale before COVID-19 Quarantine period, HRS Score (1) = Hamilton Rating Scale after COVID-19 Quarantine period, PHQ9 score (0) = Patient Health Questionnaire-9 before COVID-19 Quarantine period, PHQ9 score (1) = Patient Health Questionnaire-9 after COVID-19 Quarantine period

**Fig. 2:**
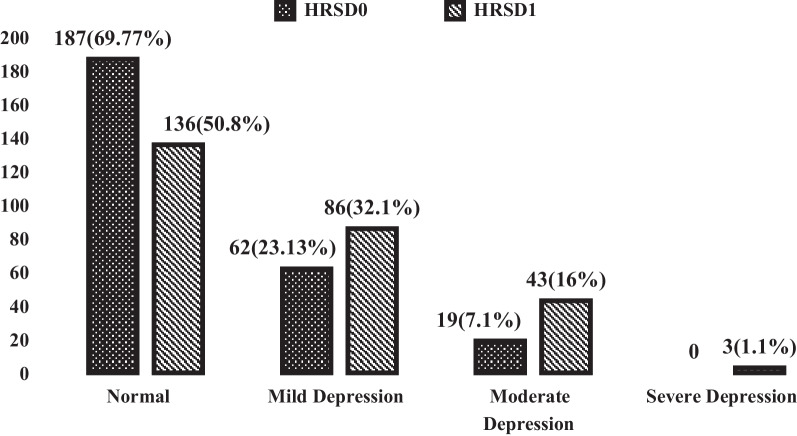
Depression degree by Hamilton Rating Scale before and after COVID-19 home quarantine period. HRSD (0) = Hamilton Rating Scale degree before COVID-19 Quarantine period, HRSD Score (1) = Hamilton Rating Scale degree after COVID-19 Quarantine period

The degree of depression severity (numbers and percent) among the students between the beginning (HRSD0) and the end (HRSD1) of the COVID-19 home quarantine period was determined according to the score of the Hamilton Depression Rating Scale (HRS). It was clear that the degree of depression increased at the end of the study (COVID-19 home quarantine period), as shown in Fig. 2.

**Fig. 3:**
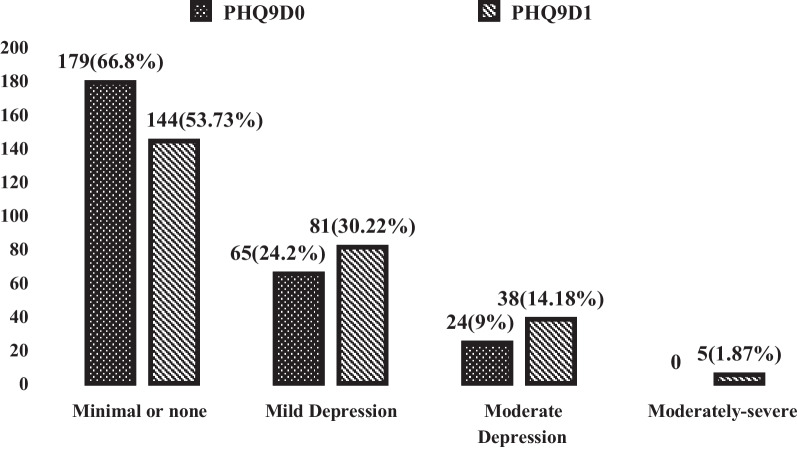
Depression degree by Patient Health Questionnaire-9 before and after COVID-19 home quarantine period. PHQ9D score (0) = Patient Health Questionnaire-9 degree before COVID-19 Quarantine period, PHQ9D score (1) = Patient Health Questionnaire-9 degree after COVID-19 Quarantine period

The degree of depression severity (numbers and percent) among the students between the beginning (PHQ9D0) and the end (PHQ9D1) of the COVID-19 home quarantine period was determined according to the Patient Health Questionnaire-9 (PHQ9D). It was clear that the degree of depression increased at the end of the study (COVID-19 home quarantine period), as shown in Fig. 3.

## Discussion

Due to the severity of COVID-19 and its negative effects on the population in general and on students in particular, this study has been conducted.

This study observed university students’ psychological depression by self-reporting methods in the COVID-19 home quarantine period. Much of the previous research on medical students’ psychological problems has applied to depression evaluation [21, 22]. Those studies included different stress resources, excluding quarantine. Study results confirmed the described depression and its complications. It additionally recommended change in the academic practices imparted by teachers that influenced the shaping and development of students’ psychological stress, which was confirmed by the presence of depression on far learning with a percent of 5% classified as major depressive disorder (MDD) as described in Korean and Spanish literature [23, 24]. That needs pressing to interfere and further studies.

This study showed the psychological depressive negative effects associated with the COVID-19 home quarantine period among pharmacy students and the factors that contribute to these effects.

At the beginning of the COVID-19 home quarantine, the percentages of the normal, non-depressed students in the studied sample were (69.77%) and (66.8%) and the percentages of the depressed students were (30.23%) and (33.2%) according to (HRS) and (PHQ9), respectively. After the COVID-19 home quarantine, the percentages of the normal, non-depressed students in the studied sample were (50.8%) and (53.73%) and the percentages of depressed students were (49.2%) and (46.27%) according to (HRS) and (PHQ9), respectively. Another study showed that 35.33% of university students had signs of anxiety and 72.93% of depression, although to a mild degree during the COVID-19 pandemic [25]. A preceding study revealed how young people, in particular university students, are more liable to psychological distress in pandemic disasters [26]. This suggests that the pandemic augmented common mental health conditions among the population, with a prevalence of anxiety and depression of about 32.9 and 35.3% in Asia and 23.8 and 32.4% in Europe, respectively [27].

This study reported a significant difference in scale scores in relation to gender (being a female) many studies of medical students have reported that female students experience more depression, anxiety, and stress compared to male students [28, 29]. But many studies reported no gender difference in the occurrence of depression among medical students [30]. In contrast, another study reported that male students are more prone to depression than female students [31]. A study proved an association between gender and anxiety level in which females more likely to develop anxiety symptoms due to health emergencies and obligatory quarantine than their male colleagues [32]. This association is debatable since other studies reported greater anxiety scores in males [33]. This change may be the result of cultural factors and gender-related attitudes and behaviors.

This study suggested that the high levels of psychological stress in females may be since female medical students are more competitive, tend to be more concerned about their grades and their performance, and they tend to exaggerate their sadness and engage in less exercise. Also, it is multifactorial, including biological, sociocultural, or varying combinations of each.

This study showed a significant difference in both depression scale scores that were used for family income categories, as the higher the family income, the lower the depression score and vice versa. This result is consistent with another study that observed a positive relationship between family income and a youth’s symptoms of depression and anxiety [34].

Regarding parental marital status; students residing with a father and mother in a stable family life have significantly lower scores of depression, and another study reported that men and women who have had a parental divorce since they were children are probably more depressed than those who did not [35].

In addition, the physical index showed a significant difference in both the depression scale scores that were used, where previous studies have shown that regular exercise reduces the incidence of depression in physiological and psychological ways. Regular practice of sport affects levels of the monoamine and endorphin neurotransmitter systems; notably, monoamines are depleted in depressed patients [36]. Psychologically exercising improves self-esteem and self-perception improves through self-actualization and benefit the satisfaction of an extended social network [37]. The level of physical activity during the quarantine acted as a defensive factor against psychological distress. The positive effect of physical activity on mental well-being has been widely shown in many studies [38, 39]. Recent studies revealed that exercising and physical activity during quarantine improves both mental and physical health [39–41], especially for younger people.

Last academic performance in this study has no significant difference in both depression scale scores, this is due to the fact that concern about the disease takes everyone’s attention.

Sleep time in this study showed a significant difference in both depression scale scores that were used. Students who sleep < 6 h per day and then > 8 h per day have more depression scores than students who sleep 6–8 h per day. These results are consistent with another study done in China [42].

College students have a high rate of mental health problems was clear in this study’s results. Suicide prevalence also was very high, according to the WHO proposal in 2005 [43] that was terrible results and must be put into consideration. Many participants expressed the opinion that the number of students with mental health problems was increasing and that the severity of their problems was also increasing. There was widespread agreement that the levels of stress were very high in the medical student population [44, 45].

The reason for the high percentage of symptoms being reported by pharmacy students could be a result of the obstacles in the process of learning, rigid practical work in addition to the culture of facing pandemics as a developing country. It is important for academic staff to be aware of the presence of these symptoms in their students. There is a great demand for this in colleges. Undergraduate pharmacy students should understand what is required of them and adapt as quickly as possible.

## Strengths/limitations

This study is considered one of the first studies that clarifies the negative psychological effects of COVID-19. The use of two different types of psychological scales is the strength of this study. As this is a unicenter study, it will be difficult to generalize the results. Because the study results were based on self-report by the participants, there is a possibility that some of the points in the scales that have been used may not have been fully understood by the students.

## Conclusion

The restriction of student movement negatively affects their psychological state and mental health, leading to depression and other morbidities.

The prevalence of psychological depression due to worry about COVID-19 during the home quarantine period among college students was high in this study. Other psychological stress problems may be associated with depression, such as suicide, guilt, insomnia, stomatitis, and agitation. Hopefully, these conditions can be stopped to prevent psychological morbidity among the college of pharmacy students and all students in general. It is clear here the important role played by the family as well as the universities in overcoming this crisis through psychological support for students and communicating with them directly with parents or online with universities for prevention, early detection, and early treatment of such cases of depression. A larger study should be done to confirm these outcomes.

## Data Availability

The datasets generated during and/or analyzed during the current study are available from the corresponding author on reasonable request.
